# Analysis of genome instability and implications for the consequent phenotype in *Plasmodium falciparum* containing mutated MSH2-1 (P513T)

**DOI:** 10.1099/mgen.0.001003

**Published:** 2023-04-21

**Authors:** Hajime Honma, Nobuyuki Takahashi, Nobuko Arisue, Tomohiko Sugishita

**Affiliations:** ^1^​ Section of Global Health, Division of Public Health, Department of Hygiene and Public Health, Tokyo Women’s Medical University, 8-1 Kawada-cho, Shinjuku, Tokyo 162-8666, Japan; ^2^​ Department of International Affairs and Tropical Medicine, Tokyo Women’s Medical University, 8-1 Kawada-cho, Shinjuku, Tokyo 162-8666, Japan

**Keywords:** *Plasmodium falciparum*, MutS homologue 2, PfMSH2-1, mutator, mutation accumulation test, malaria

## Abstract

Malarial parasites exhibit extensive genomic plasticity, which induces the antigen diversification and the development of antimalarial drug resistance. Only a few studies have examined the genome maintenance mechanisms of parasites. The study aimed at elucidating the impact of a mutation in a DNA mismatch repair gene on genome stability by maintaining the mutant and wild-type parasites through serial *in vitro* cultures for approximately 400 days and analysing the subsequent spontaneous mutations. A P513T mutant of the DNA mismatch repair protein PfMSH2-1 from *Plasmodium falciparum* 3D7 was created. The mutation did not influence the base substitution rate but significantly increased the insertion/deletion (indel) mutation rate in short tandem repeats (STRs) and minisatellite loci. STR mutability was affected by allele size, genomic category and certain repeat motifs. In the mutants, significant telomere healing and homologous recombination at chromosomal ends caused extensive gene loss and generation of chimeric genes, resulting in large-scale chromosomal alteration. Additionally, the mutant showed increased tolerance to N-methyl-Nʹ-nitro-N-nitrosoguanidine, suggesting that PfMSH2-1 was involved in recognizing DNA methylation damage. This work provides valuable insights into the role of PfMSH2-1 in genome stability and demonstrates that the genomic destabilization caused by its dysfunction may lead to antigen diversification.

## Data Summary

The sequence data generated during the current study are deposited in DDBJ/EMBL/GenBank databases under the BioProject accession number PRJDB12929. The synthetic *bsd* gene sequence was deposited in DDBJ/EMBL/GenBank databases under the accession number LC743844. Supplementary data is available with the online version of this article.

**Impact Statement**

The most dreadful malarial parasite, *Plasmodium falciparum*, develops drug resistance and exhibits host immune evasion, making malaria control more complicated. Understanding the mechanisms underlying genome stability is important because mutations accumulating during cell proliferation are the driving force behind the creation of such undesirable phenotypes/traits. In some pathogens, defects in the DNA mismatch repair (MMR) system increase mutation rates, resulting in accelerated development of drug resistance; however, this association remains unclear in *P. falciparum. Msh2-1* is one of two *MutS homologue 2* paralogues in the *P. falciparum* genome and is involved in MMR. We herein generated a novel mutator parasite by introducing a proline-to-threonine substitution at position 513 in *pfmsh2-1*. High-throughput genome sequencing of wild-type and mutator parasites revealed that insertion/deletion mutation rate in the mutator parasite was significantly higher than that in the wild-type parasite, but that base substitution rates were comparable between them. Further, PfMSH2-1 deficiency destabilized sub-telomeric regions, causing massive gene loss and generation of new chimeric antigen genes. This study suggests that PfMSH2-1 deficiency does not promote the acquisition of point mutation-induced drug resistance, but induces antigen diversification and thus contributes to conferring immune evasion ability on *P. falciparum*.

## Introduction

Malaria deaths declined steadily between 2000 and 2019 but increased by 12 % (~627 000) in 2020 compared to that in 2019 due to service disruptions during the COVID-19 pandemic [1]. Therefore, malaria is a significant public health concern that is worsening in endemic areas. Malarial parasites exhibit extreme genetic/genomic plasticity, which makes combating malaria difficult. Of the five human malarial parasites, the most virulent species, *Plasmodium falciparum*, has a highly AT-rich nuclear genome, mainly consisting of a stable core domain, containing single-copy housekeeping genes, and a highly variable sub-telomeric domain with multigene families encoding various surface antigens, such as *var*, *stevor* and *rifin* [2]. Owing to single nucleotide polymorphisms and/or amplification of genes responsible for drug resistance, *P. falciparum* has developed resistance against almost all known antimalarials [3, 4]. Additionally, *P. falciparum* exhibits extensive antigenic variation to evade host immunity, facilitating long-term persistent infection and recurrence. Antigenic variation is dependent on the sequence polymorphism of antigen genes caused by ectopic recombination among genes of a multigene family during meiosis and mitosis [5–8].

In general, low mutation rates in organisms are achieved through the combined action of DNA polymerases and the DNA mismatch repair (MMR) machinery [9]. Defects in any of these can result in mutator phenotypes with high mutation rates. We previously reported a mutator malarial parasite termed PbMut possessing a 36.5-fold higher base substitution rate than did the wild-type, generated after the loss of proofreading activity of DNA polymerase δ in *P. berghei* ANKA [10, 11]. An attempt to investigate the drug-resistance mechanism using a *P. falciparum* mutator line, generated on the same basis as PbMut, has also been reported [12], demonstrating the usefulness of the malaria mutator as a genetic tool. Various proteins are involved in MMR, and mutant strains containing defects in any of these proteins easily acquire resistance to antibiotics [13, 14] and antifungals [15, 16]. In *P. falciparum*, a mild mutator phenotype has been linked to a mutation in the MMR protein PfMLH1 [17]. An association between drug-resistance acquisition and defective MMR has been suggested [18, 19]; however, the contribution of MMR to genome stability and evolutionary mechanisms that give rise to undesirable phenotypes, such as host immune evasion and drug resistance, remain to be elucidated.

MutS homologue 2 (MSH2) is a major MMR protein that recognizes DNA mismatches and is highly conserved in eukaryotes. *Plasmodium* is the only eukaryotic taxon with two *msh2* paralogs (*msh2-1* and *msh2-2*, 64 % similarity) [20]. In a previous study, microsatellite instability was suggested in the *pbmsh2-2*-disrupted strain of *P. berghei* NK65 [20]. Researchers also attempted to disrupt *pbmsh2-1* without any success [20]. However, no study has been conducted to assess the role of *msh2-1* in the maintenance of genome stability. We generated a PfMSH2-1^P513T^ mutant of *P. falciparum* using the CRISPR/Cas9 technology. Proline at position 513 in PfMSH2-1 is a highly conserved site across organisms and corresponds to P622 and P642 in humans and the budding yeast MSH2, respectively (Fig. S1A, available with the online version of this article). The P513T mutation at this locus is presumed to be associated with an increased mutation rate [21]. To elucidate the influence of PfMSH2-1 defects resulting from the P513T mutation on genome stability, mutant and wild-type parasites were maintained by serial *in vitro* passage for approximately 400 days, and spontaneous mutations were evaluated using high-throughput sequencing (HTS). Parasites were exposed to an alkylator and tested for resistance to examine the response of PfMSH2-1 to DNA damage. We believe that this research will be useful for understanding the contribution of PfMSH2-1 in maintaining nuclear genome stability and the consequent phenotypes caused by its dysfunction.

## Methods

### Plasmid construction

The plasmids to be edited using CRISPR/Cas9 were constructed using pL6_eGFP and pUF1_Cas9 [22]. The *ydhodh* gene, integrated into pUF1_Cas9, was replaced with a synthetic *bsd* gene cassette with a sequence optimized for *P. falciparum* codon usage on Geneious R10 (https://www.geneious.com). The *bsd* gene cassette was amplified from a synthetic plasmid using PCR and the BSD-BamHI-F/BSD-HindIII-R primers (Table S1), followed by double digestion with BamHI and HindIII. The DNA fragment was integrated into BamHI/HindIII-digested pUF1_Cas9 using a DNA Ligation Kit <Mighty Mix> (TaKaRa Bio, Shiga, Japan), generating pUF1_Cas9_BSD. pL7_PfMSH2-1^P513T^ (Fig. S1B), with a guide RNA sequence leading Cas9 to the *pfmsh2-1* gene region and a homologous sequence to fix the double-strand break (DSB) by homologous recombination (HR) was constructed from pL6_eGFP in a two-step process. First, the guide RNA cassette (Table S1) designed by chopchop v2 [23] was introduced into pL6_eGFP after digestion with BtgZI to generate an intermediate plasmid. Second, the SpeI/AflII-digested intermediate plasmid and homology arms that were amplified from 3D7 genomic DNA by performing PCR with pCC1box1-SpeI-P513T-F1/Mut-P513T-R1 and Mut-P513T-F2/pCC1box1-AflII-P513T-R2, were assembled to generate pL7_PfMSH2-1^P513T^ (Fig. S1B and Table S1). The assembly steps used to construct pL7_PfMSH2-1^P513T^ were performed using the In-Fusion HD cloning kit (Clontech, CA, USA).

### *P. falciparum* 3D7 culture and CRISPR/Cas9 genome editing

*P. falciparum* 3D7 was statically cultured in RPMI-1640 medium with l-glutamine (Thermo Fisher Scientific, Waltham, MA, USA) supplemented with 25 mM HEPES, 0.05 mg ml^−1^ hypoxanthine, 10 µg ml^−1^ gentamycin, 0.2 % sodium bicarbonate, 0.25 % AlbuMAX II (Thermo Fisher Scientific), 5 % human AB plasma-derived serum [24] and human A+erythrocytes. Parasite cultures were maintained using the standard candle jar method [25] or the Anaeropack️ plas system (5 % O_2_, 5 % CO_2_; Mitsubishi Gas Chemical Company, Tokyo, Japan) [26].

Ring-stage synchronized culture (parasitaemia >5 %) with 5 % sorbitol treatment was electroporated using a gene pulser (Bio-Rad, Hercules, CA, USA) at 0.31 kV, 960 µF. Drug selection with 50 ng ml^−1^ pyrimethamine and 2.5 µg ml^−1^ blasticidin S was applied to transfected cultures 16–20 h post-electroporation. No parasites were observed under a microscope after 1 week; thereafter, the addition of blasticidin S was continued until parasite growth could be observed under a microscope. We then confirmed the introduction of the P513T mutation into the *pfmsh2-1* gene using Sanger sequencing and obtained the PfMSH2-1^P513T^ clone by limiting dilution.

We maintained six mutation accumulation (MA) lines for each Mut and WT based on the limiting dilution approach (Fig. S2). Parasites were seeded into 96-well plates with 0.5 parasitized red blood cells (pRBCs) per well, 200 µl malaria complete media (MCM), and 2 % haematocrit. Approximately 2 weeks later, parasite growth was evaluated using a *Plasmodium* lactate dehydrogenase activity assay [27, 28], and one of the parasite cultures, randomly chosen from positive wells, was transferred into a 12-well culture plate with 4 ml MCM and 4 % haematocrit and cultured for approximately 5 days. Thereafter, a part of the culture was used for the next round of limiting dilutions and culture, and the rest was used for cryopreservation. Eventually, MA clones were maintained for 399–441 days.

### Estimation of *pfmsh2-1* and *pfmsh2-2* gene expression

pRBCs at the late trophozoite stage were lysed with 0.075 % saponin. The saponin-lysed pRBCs were harvested in RNAiso Blood (TaKaRa Bio) and subsequently subjected to the FastGene RNA Premium Kit (Nippon Genetics, Tokyo, Japan) to extract total RNA. cDNA samples were prepared using the High-Capacity cDNA Reverse Transcription Kit (Thermo Fisher Scientific). Quantitative real-time PCR assays were performed with two technical replicates for each of the three biological replicates for both Mut and WT using the KOD SYBR qPCR Mix (Toyobo, Osaka, Japan) and primers listed in Table S1 on a StepOne system (Thermo Fisher Scientific) according to the manufacturer’s instructions. We used *pfact1* and *pffbpa* as internal controls [29]. The relative expression levels were calculated as a ratio of the target gene expression to that of the internal control.

### Growth test

Synchronized pRBCs with 5 % sorbitol treatment were inoculated in 4 ml MCM at an initial parasitaemia of 0.01 and 2% haematocrit. Parasite density was evaluated every 12 h by counting the number of infected erythrocytes per 3 000 erythrocytes based on Giemsa-stained thin blood smears. Growth assays were performed for each Mut and WT (six replicates). Growth data were fitted to the logistic equation using GrowthCurver [30] to estimate the growth rate and maximum possible population size.

### DNA extraction and HTS

Genomic DNA of the MA clones and their progenitors was extracted using a QIAamp DNA Mini Kit (Qiagen, Hilden, Germany). Library preparation was conducted using the NEBNext Ultra II DNA Prep Kit for Illumina (New England Biolabs, MA, USA) and sequenced using Illumina HiSeq X Ten. To analyse chromosome ends in detail, long read sequence data were also obtained from samples in which large deletions were observed in sub-telomeric regions using Illumina reads. Genomic DNA was extracted using GenFind V3 (Beckman Coulter, Brea, CA, USA) and sequenced using Oxford Nanopore Technologies (ONT) MinION.

### DNA sequence data analysis

To analyse mutations accumulated during long-term passage cultures, the paired-end reads were aligned to the *P. falciparum* 3D7 reference genome sequence downloaded from PlasmoDB (https://plasmodb.org/plasmo/app/downloads/release-41/Pfalciparum3D7/) using BWA-MEM v0.7.17 [31] after applying adapter and quality trimming with Trim Galore v0.6.5 (https://github.com/FelixKrueger/TrimGalore). Duplicate sequences were removed using Picard tools v2.22.9 (https://broadinstitute.github.io/picard/index.html). The base quality score was recalibrated in five rounds using the Genome Analysis Tool Kit (GATK) v4.1.7.0, based on variants called using GATK4-HaplotypeCaller [32]. Subsequently, single nucleotide variants (SNVs) and insertion/deletions (indels) were detected from the alignments using the Fermikit v0.14.dev1 [33], GATK4-HaplotypeCaller [32], Platypus variant caller v0.8.12 [34] and VarScan2 v2.4.4 [35]. SNV and indel data were merged using BCFtools v1.8 [36]. Structural variations (SVs) were detected from alignments using Delly v0.8.3 [37], Fermikit v0.14.dev1 [33], LUMPY v0.3.0 [38], Manta v1.6.0 [39] and Wham v1.7.0 [40] and subsequently, the data were merged using survivor v1.07 [41]. Detected variants were confirmed after visual inspection of mapping data using Integrative Genomics Viewer (IGV) [42]. When we found SNVs and indels supported by a few reads, we further validated them using Sanger sequencing (Table S1). Actually, we attempted 33 Sanger analyses and 28 indels were validated (Table S5). Finally, we determined the accumulated mutations in MA lines by removing the variant sites identified in progenitor clones. The detected variants were annotated using SnpEff v4.3.1t [43].

The ONT reads were assembled using Canu v2.1.1 [44]. The assemblies were corrected with Racon v1.4.20 [45] up to ten times, and the most optimal assemblies were chosen according to the scores calculated by busco v5.0.0 [46]. The assemblies and raw ONT reads were mapped to the reference genome sequence using Minimap2 v2.17 [47]. Through a manual review of the mapping data on IGV, the Canu assemblies mapped to the sub-telomeric regions containing deletions were identified and retrieved. Canu assembly and the sequence of the corresponding sub-telomeric region were realigned using mafft v1.4.0 [48] to determine whether telomere healing or HR occurred. In the case of HR, we identified the bound sequence origin by searching it using blastn v2.9.0 [49] and reviewing the SV data. Plots were generated using Circos software to display genome variations [50]. The programme settings used in this study are shown in Table S3.

### False-negative rate of variant call pipeline used in this study

We estimated the false-negative rate by simulating synthetic mutations in Illumina mapping data. Approximately 1 000 SNVs were introduced into the core-genome regions [51] using the addsnv.py script in BAMsurgeon [52]. Likewise, we generated mapping data containing synthetic indel mutations. First, we randomly chose 1 000 short tandem repeats (STRs) loci across core genome regions [51, 53]. Next, we introduced indel mutations consisting of the repeat motif for each STR into the loci using the addindel.py script in BAMsurgeon [52]. Paired-end reads containing *de novo* mutations were retrieved from the alignments using SAMtools v1.9 [36] and SeqKit v0.14.0 [54]. We aligned the new reads to the reference sequence and called the mutations using the pipeline described above. We compared the SNV and indel calls with the expected genotypes and calculated the false-negative rates, as described previously [55].

### Calculation of the mutation rate

The base substitution rate (*μ*_bs_) was calculated according to the equation: *μ*_bs_ = *m* / (*n*×AC), where *m* is the number of SNVs detected, *n* is the number of nucleotide sites mapped by ≥2 reads, and AC corresponds to the number of asexual cycles, which was considered as half the number of passage days because the duration of asexual multiplication in erythrocytes of *P. falciparum* was approximately 48 h. The indel mutation rate was calculated by replacing *m* and *n* in the above equation with the number of indels and STRs with an average depth of coverage of ≥2. Similarly, genomic category, allele size and repeat motif-dependent indel mutation rates were calculated. The depth of each nucleotide site and the average depth of coverage for each STR locus were estimated using Mosdepth v0.3.1 [56]. We corrected the mutation rates using false-negative rates estimated from synthetic mutations [57, 58].

### Tolerance test to alkylator

To investigate the difference in tolerance to N-methyl-Nʹ-nitro-N-nitrosoguanidine (MNNG) between Mut and WT, ring-stage synchronized pRBCs with 5 % sorbitol treatment were collected and placed in MCM in the absence and presence of 1.5 µg ml^−1^ or 3 µg ml^−1^ MNNG at 2 % haematocrit and an initial parasitaemia of 0.3 %. After 24 h, the medium was replaced with normal MCM. Parasite growth and cell morphology were observed under a microscope 2–5 days post-exposure. The relative growth rate was calculated by comparing the parasitaemia of the MNNG-exposed group to that of the control group. This test was performed with four replicates.

### Statistical analysis

All statistical tests were conducted using the R software [59]. Statistical significance was set at *P*<0.05. Graphs were generated using the ggplot2 package [60]. We fitted the random-intercept logistic regression model for our indel data using the glmer() function in the lme4 package [61] and the lmerTest package [62] to calculate *P*-values of fixed effect estimates (Tables S3 and S4).

## Results

### Generation of the PfMSH2-1^P513T^ mutant from *P. falciparum* 3D7 and serial *in vitro* culture passage

Amino acid substitutions that contribute to increased mutation rates have been identified in the budding yeast MSH2 [21]. Based on a previous study [21], we attempted to generate mutants (R414L, P513T and G565R) of PfMSH2-1 associated with high mutation rates using the CRISPR/Cas9 technology, but succeeded in generating only a P513T mutant (Fig. S1B). In this study, the parental 3D7 strain was denoted as WT and the P513T mutant as Mut. We obtained progenitor clones, WT P0 and Mut P0, by cloning with a limiting dilution of *P. falciparum* 3D7 and PfMSH2-1^P513T^ mutant for the MA test. We confirmed whether the *cas9* gene was still present in Mut P0 using PCR, but the result was negative (Fig. S1C). Real-time PCR analysis showed no significant differences in the expression levels of *pfmsh2-1* and *pfmsh2-2* between P0 clones of Mut and WT parasites (Fig. S1D), suggesting that PfMSH2-1^P513T^ does not affect the expression of these genes. Thin blood smears were microscopically observed every 12 h to compare the growth of P0 clones (Fig. S3). There was no significant difference in growth rate between Mut and WT (*P*=0.84; Welch’s *t*-test), but maximum parasitaemia was significantly higher in WT than in Mut (*P*=0.005099; Welch’s *t*-test). To investigate the spontaneous mutation characteristics during the long-term passage, P0 clones were divided into six parallel MA lines, and continuous *in vitro* culture passage was conducted for approximately 400 days based on the limiting dilution method (Fig. S2).

### Overview of mutations and mutation rates

Genomic DNA extracted from P0 and MA clones was sequenced by an Illumina sequencer. Illumina short reads were mapped to the *P. falciparum* 3D7 reference genome sequence, and spontaneous mutations were detected as described in Methods. Table S5 summarizes the genome sequence data for this study. We preliminary revealed the difference of genotypes between Mut P0 and WT P0, and found no significant changes that would affect mutation rate, except for P513T in PfMSH2-1 (Table S6). The number of detected SNVs per clonal lineage, excluding the mutation cluster near SVs, was comparable between the Mut (3–8 SNVs) and WT (2–6 SNVs); however, the number of indels was higher in Mut (79–115 indels) than in WT (5–16 indels) (Tables S2 and S7). Most indels occurred in microsatellite loci, also referred to as STRs, and in minisatellite loci. The base substitution rate (/bp/asexual cycle) (mean±se) was not significantly different between the Mut and WT parasites (Table 1). In contrast, the indel mutation rate at STRs (/locus/asexual cycle) in the core genome defined as repeat arrays with least three repeat 1–9 bp units ranging from 10 to 70 bp in length [51, 53] was 9.9-fold higher in Mut than in WT (Table 1). The indel mutation rate at minisatellite loci (/locus/asexual cycle) in the core genome defined as repeat arrays with at least three repeat 10–30 bp units ranging from 30 to 1000 bp in length [51, 53] was 4.8-fold higher in Mut than in WT (Table 1).

**Table 1.** Mutation rates estimated in this study and reported in previous studies

Strain	Base substitution rate*		Indel mutation rate at STRs*		Indel mutation rate at minisatellites		Reference
	Mean±se (×10^−10^)	*P*-value†	Mean±se (×10^−7^)	*P*-value†	Mean±se (×10^−7^)	*P*-value†	
Mut	7.65±1.85	0.48	37.1±2.3	1.2e-05	24.0±4.69	0.02	This study
WT (3D7)	5.98±1.34		3.76±0.6		5.00±5.00		This study
3D7	5.23‡		na		na		8, 53
3D7	3.18±0.74		4.43±0.37		na		53
3D7	2.10		4.45±0.97‡		na		53, 68

*Mutation rates corrected for false negative rates.

†*P*-values of Welch’s *t*-test.

‡These values were estimated in reference 53 based on data reported in references 8 and 68. na, not available.

### Estimation of the false-negative rate of the variant calling pipeline

To evaluate the performance of the analysis pipeline used in this study, we generated mapping data that included artificially introduced variants using BamSurgeon [52] and called variants using the same pipeline. The false-negative rates were 0.004 and 0.138 for base substitution and indel detection, respectively. These values are comparable to or lower than those reported in previous studies [55, 57, 58]; therefore, the power of the analysis pipeline used in this study was sufficiently high.

### Effect of STR characteristics on mutability

**Fig. 1.**
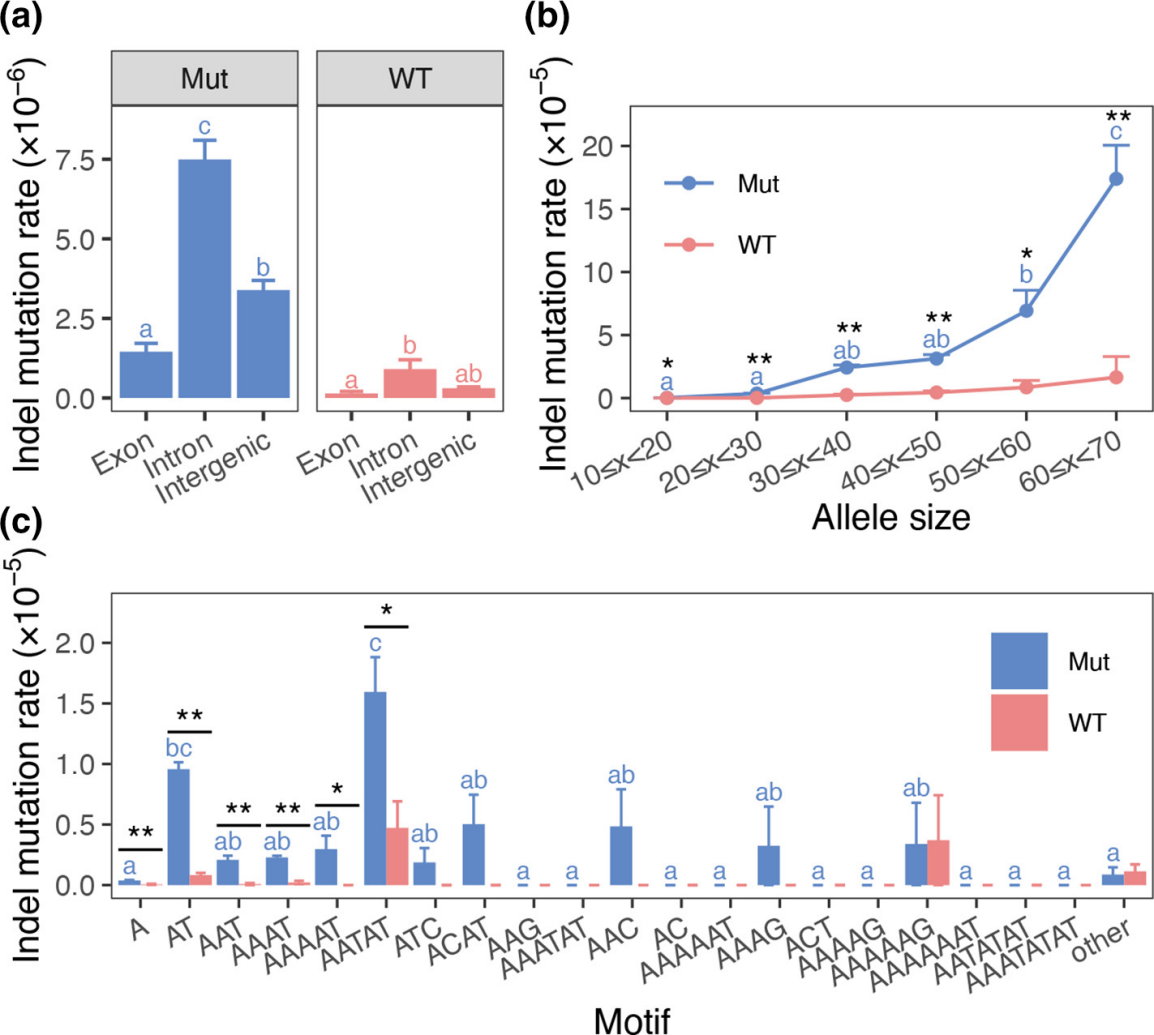
Mutation rates estimated with the indel data obtained from MA clones of Mut and WT. (**a**) Genomic category-dependent mutation rates. (**b**) Allele size-dependent mutation rates. (**c**) Repeat motif-dependent mutation rates. There was a statistically significant difference between means with different alphabets (*P*<0.05; Tukey–Kramer test) when compared within the same parasite-type clones. Error bars represent standard error of the mean. The asterisk indicates a statistically significant difference when comparing means between Mut and WT (^*^ *P*<0.05, ^**^ *P*<0.01; Welch’s *t*-test).

Using a large indel mutation dataset obtained in this study, we analysed the effect of STR characteristics, such as genomic region, length and repetitive motifs, on the occurrence of indels. The indel mutation rate was estimated for each genomic category and was divided into three categories: intergenic regions, exons and introns (Fig. 1a).

In the case of Mut, the indel mutation rate in introns was significantly higher than that in other genomic categories, and the indel mutation rate in intergenic regions was significantly higher than that in exons (*P*<0.05; Tukey–Kramer test). In the case of WT, the mutation rate in introns was significantly higher than that in exons (*P*<0.05; Tukey–Kramer test), but there was no difference between those in introns and intergenic regions (*P*=0.069; Tukey–Kramer test).

The mutation rate, calculated by dividing the allele size into classes of 10 bp each, increased with the allele size (Fig. 1b). In the case of Mut, the highest mutation rate was observed in the class with an allele size of ≥60 bp (*P*<0.05; Tukey–Kramer test). No significant difference in the mutation rate of WT was observed among the classes using the Tukey–Kramer test.

STRs are tandemly repeated, and some motifs are equivalent. We considered motifs to be equivalent if (1) they are reverse complementary to each other, and (2) the same repeat sequence appears after frameshift(s). The repeat motifs comprising 123 722 STRs in the *P. falciparum* core genome were classified into 458 types, and their distribution was highly skewed (Fig. S4A and Table S8). When compared with mononucleotide motifs, ‘A’ accounted for 37.7 %(46 600/123 722 STRs) of all STRs, while ‘C’ accounted for only 0.02 %(26/123 722 STRs). When compared to dinucleotide motifs, ‘AT’ accounted for 35.2 %(43 490/123 722 STRs) of all STRs, ‘AC’ for 0.27 %(329/123 722 STRs), ‘AG’ for 0.07 %(83/123 722 STRs), and ‘CG’ for <0.01 %(1/123 722 STRs). The top two motifs: ‘A’ and ‘AT’ accounted for more than 70 % of all motifs, and the top five motifs: ‘A’, ‘AT’, ‘AAT’, ‘AAAT’ and ‘AAAAT’ accounted for more than 90 % of all motifs. The distribution of repeat motifs differed among the genomic categories (Fig. S4B). Although the percentage of ‘A’ repeats differed significantly among genome categories (holm-adjusted *P*<0.05; Fisher’s exact test), they accounted for >30 % in all categories, whereas ‘AT’ repeats, which accounted for >40 % in intergenic regions and introns, were less abundant in exons (<3 %). In the exon region, STRs consisting of trinucleotide repeats, such as ‘AAT’, ‘ATC’ and ‘AAG’, accounted for a large proportion, but repeat motifs with sizes not divisible by three were scarce except for ‘A’.

ANOVA showed that the motif-dependent mutation rates (Fig. 1c) differed by motif type (Mut, *P*=3.13e-11; WT, *P*=0.0293). In Mut, the mutation rate of ‘AATAT’ repeats was significantly higher than that of other motifs, and ‘AT’ repeats also showed a significantly higher mutation rate than did many other motifs (*P*<0.05; Tukey–Kramer test). In contrast, the indel mutation rate was low in ‘A’ repeats, which exist abundantly as ‘AT’ repeats in the core genome. A comparison of the mutation rates between Mut and WT showed significant differences in the top six motifs (*P*<0.05; Welch’s *t*-test), suggesting no biassed increase in the mutation rate for any motif in Mut.

A random-intercept logistic regression model was fitted to analyse the association between mutability and STR characteristics, including length, genomic category and repeat motif. We used the STR loci where the average read depth is two or more with the most prevalent six motifs (‘A’, ‘AT’, ‘AAT’, ‘AAAT’, ‘AAAAT’ and ‘AATAT’). Both STR locus and parasite type (Mut or WT) were used as random effects. STR length, repeat motifs and introns of the genomic category were positively associated with STR mutability; however, the intergenic region of the genomic category was not statistically supported (Table 2).

**Table 2.** Random-intercept logistic regression model for predicting STR mutability

Predictors		Odds ratios [95 % CI]	*P*-value
STR length		1.14[1.13–1.15]	**< 0.001**
Genomic category (reference group, Exon)			
	Intergenic region	1.49[0.72–3.11]	0.284
	Intron	2.16[1.03–4.55]	**0.042**
Repeat motif (reference group, A)			
	AAAAT	4.73[2.12–10.58]	**< 0.001**
	AAAT	5.23[2.89–9.48]	**< 0.001**
	AAT	6.76[3.06–14.96]	**< 0.001**
	AATAT	10.30[5.12–20.75]	**< 0.001**
	AT	14.87[9.46–23.37]	**< 0.001**

### Indels in ‘AT’ repeats

**Fig. 2.**
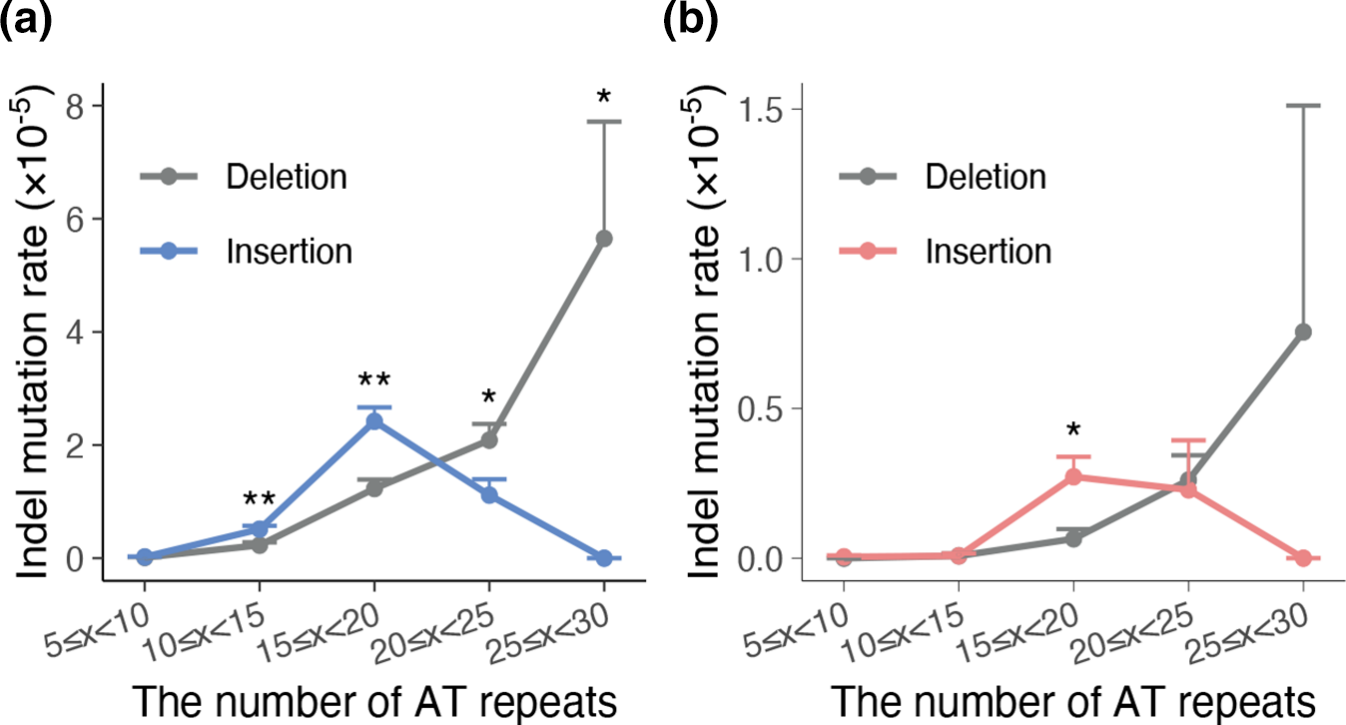
Distribution of deletion and insertion mutation rates at ‘AT’ repeats by repeat numbers. The mutation rates were calculated for each class of five ‘AT’ repeats in Mut (**a**) and WT (**b**). Error bars represent standard error of the mean. The asterisk indicates a statistically significant difference between insertion and deletion mutation rates (^*^ *P*<0.05, ^**^ *P*<0.01; Welch’s *t*-test).

There was no significant difference between the number of insertions and deletions in WT, but insertions occurred 1.4 times more than deletions in Mut (*P*=0.03227; Welch’s *t*-test) (Fig. S5A). ‘AT’ repeats were abundant in the *P. falciparum* genome and also displayed a high mutation rate; therefore, a large number of indels spontaneously accumulated in ‘AT’ repeats during the long-term passage (Fig. S5B). When the insertion or deletion mutation rates were calculated for each class of repeat number, the deletion mutation rate tended to increase with the number of repeats, whereas the insertion mutation rate showed a unimodal distribution with a peak in the class of 15‒20 repeats (Fig. 2). The insertion mutation rate was higher when the number of repeats was <20, but the indel mutation rate was higher when the number of repeats was ≥20. This tendency was observed in both Mut and WT parasites and was more pronounced and statistically significant in Mut (*P*<0.05; Welch’s *t*-test).

### Destabilization of sub-telomeric regions in Mut

**Fig. 3.**
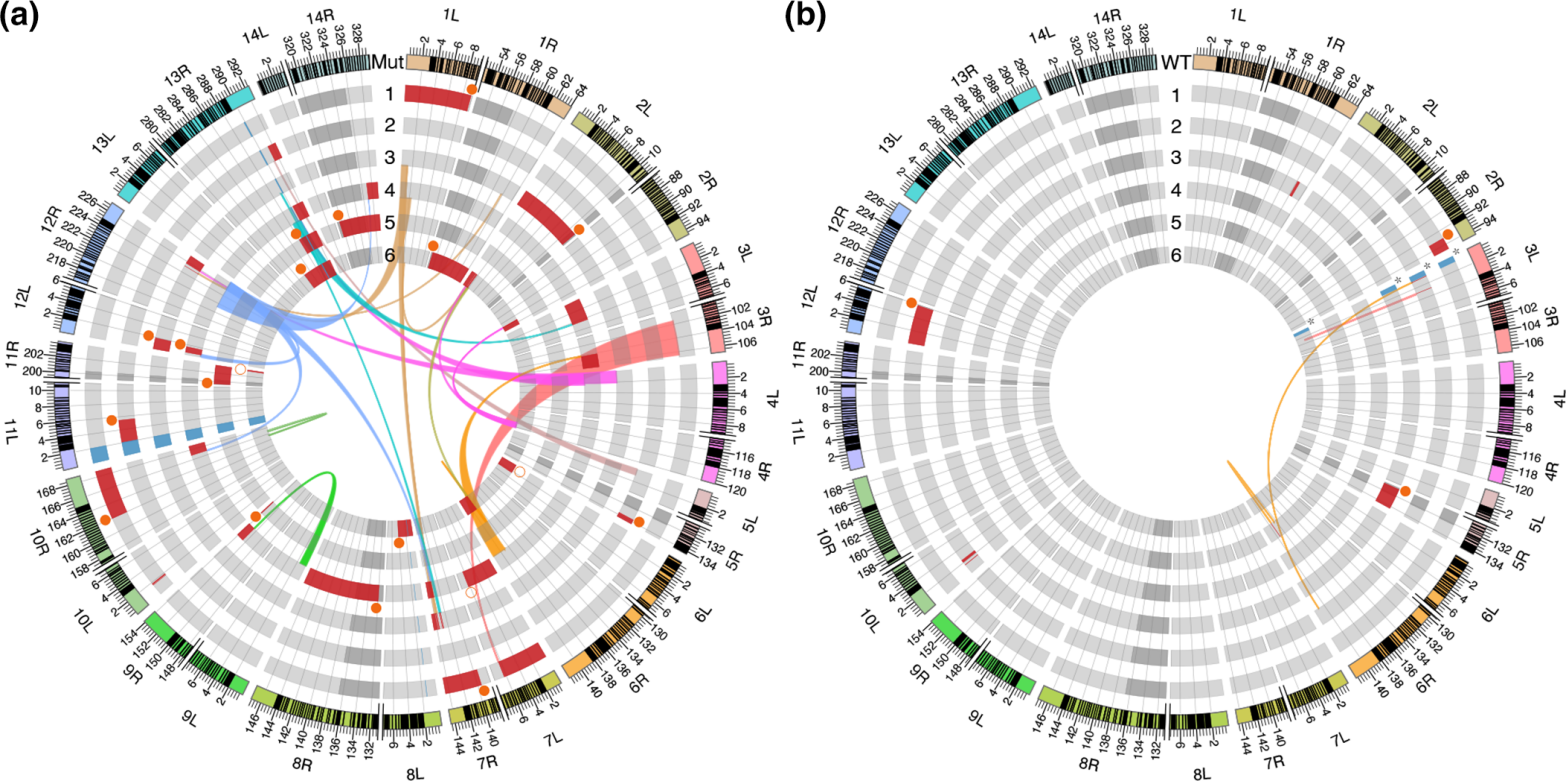
Circos plots depicting genome variations in Mut (**a**) and WT (**b**). The outermost track shows the 14 chromosome end regions of *Plasmodium falciparum* in which the letters ‘L’ and ‘R’ denote left and right ends, respectively. One scale label indicates 4 kbp. The locations of genes are shown with black lines. The inner six rings represent chromosome ends of MA clones represented as follows: intact sub-telomere regions (light grey), intact core genome regions (grey), large deletions (dark red), and large deletions shared with progenitor (light blue) or some MA clones (light blue with asterisk). Solid orange circles indicate breakpoints repaired by telomere healing. Ribbons show homologous recombination, and the colour indicates the chromosome from which the donor region to repair was derived. Open orange circles indicate breakpoints where the repair mechanism was not identified.

We observed several chromosome end regions where no Illumina short reads were mapped, except for three WT clones, WT4–6. At the 14 chromosome ends of each MA clone, except for a few common sub-telomeric deletions in some clones probably due to insufficient cloning procedure in making P0 clones, deletions of original sub-telomeric regions occurred significantly more frequently in Mut than in WT (33 locations vs. 3 locations, *P*=8.161e-09; Fisher’s exact test) (Fig. 3 and Table S9). The deleted length of the original chromosomal ends varied from 1.7 to 15 kbp, and the total length of deleted regions (mean±se) was significantly larger in Mut (233±47.7 kbp) than in WT (18.5±9.97 kbp) (*P*=0.005737; Welch’s *t*-test) (Fig. S6). To examine how these regions were repaired, we conducted SV detection by mapping data using the Illumina reads (Table S10). Additionally, we obtained ONT long reads to generate high-quality assemblies and compared them with the reference genome sequence. Although we could not determine the repair mechanism in some regions, the deleted regions were replaced by other sub-telomeric or telomeric sequences using intra/inter-chromosomal recombination or telomere healing, respectively (Fig. 3 and Table S9). As a result of sub-telomeric deletions, we observed the loss of many genes coded on the original chromosome ends (Table S11). For example, the histidine-rich protein 2 (*hrp2*) gene (PF3D7_0831800), which is widely used as a target for malaria rapid diagnostic tests (RDTs), already possessed a large deletion in Mut P0 and was completely replaced by the telomeric sequence in Mut4 during long-term passage (Fig. S7A–C). In addition, most of *hrp3* (PF3D7_1372200) in Mut6, the product of which cross-reacts with antibodies of HRP2 [63, 64], was replaced by telomeric repeats (Fig. S7D, E). Furthermore, HR events occurring on chromosomes 3 and 14 in Mut4 generated chimeric genes. Results of Sanger sequencing demonstrated that the HR event between two *var* (PF3D7_1373500 and Pf3D7_030010) produced a chimeric *var*, and the HR event that occurred between two *stevor* (PF3D7_1479500 and PF3D7_1254300) resulted in a chimeric *stevor* (Fig. S8A–E). Notably, these recombination events retained their reading frame.

### MNNG tolerance test

**Fig. 4.**
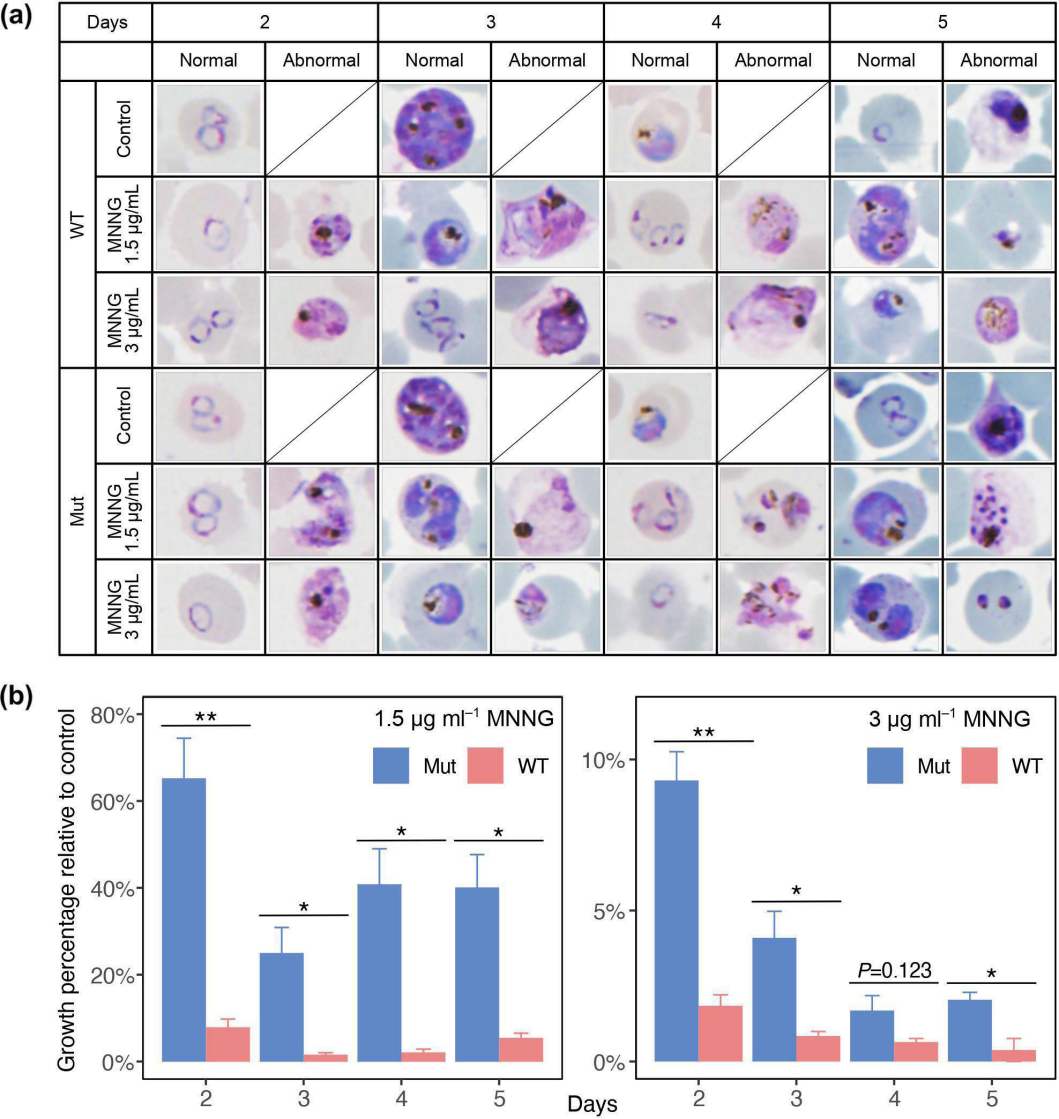
Susceptibility of Mut and WT to N-methyl-N’-nitro-N-nitrosoguanidine (MNNG). The ring-stage synchronized parasitized RBCs (pRBCs) were grown in malaria complete medium with 0 µg ml^−1^ (control), 1.5 µg ml^−1^ and 3 µg ml^−1^ MNNG for 24 h, and microscopic observation was performed 2‒5 days post-exposure. (**a**) The representative cell morphology of MNNG-treated and untreated parasites. The parasites in the control group developed normally, while those in the 1.5 µg ml^−1^ and 3 µg ml^−1^ MNNG-treated groups showed abnormal morphology with reddish colour and indistinct cytoplasm and nuclei. This abnormal morphology was not observed in the control group. Conversely, abnormal morphology of parasites with cell shrinkage due to overgrowth was observed on day 5 in the control group. (**b**) The pRBCs with normal morphology were counted under a microscope 2–5 days post-exposure. The percentage survival of the MNNG-exposed group relative to that of the control group was assessed and plotted. Error bars represent standard error of the mean. The asterisk indicates a statistically significant difference when comparing means between Mut and WT (^*^ *P*<0.05, ^**^ *P*<0.01; Welch’s *t*-test).

MSH2 is also known to be involved in response to DNA damage caused by genotoxic agents [65]; however, it is unclear whether *P. falciparum* MSH2-1 also plays a role in recognizing base mispairing resulting from DNA damage. We exposed Mut and WT parasites to MNNG and compared their growth *in vitro*. MNNG gives rise to O^6^-methylguanine (O^6^-MeG), and thymine can be mis-incorporated into O^6^-MeG during DNA replication. Defective MMR is associated with increased tolerance to MMNG due to the loss of a ‘futile repair cycle’ in which MMR fails to remove O^6^-MeG and reinsertion of cytosine or thymine opposite the lesion occurs [66]. Abnormal parasite cells of reddish colour with indistinct cytoplasm and nuclei were observed in the MNNG-treated groups (Fig. 4b). On day 5, abnormal morphology of parasite cells due to overgrowth [67] was also observed in the control group, which was not exposed to MNNG, but it appeared to differ in morphology from the cell impairment caused by MNNG exposure (Fig. 4a). We calculated the relative growth percentage of the MNNG-exposed group relative to that of the control group. Stronger cytotoxic effects were observed in the 3 µg µl^–1^ exposure group than in the 1.5 µg µl^–1^ exposure group (Fig. 4b). In the 1.5 µg µl^–1^ exposure group, the relative growth percentage was significantly higher in Mut than in WT during the entire observation period (*P*<0.05; Welch’s *t*-test). In the 3 µg µl^–1^ exposure group, significantly higher relative growth rates in Mut were observed, except on day 4 post-exposure (*P*<0.05; Welch’s *t*-test) (Fig. 4b). These results show that Mut is more tolerant to MNNG exposure than WT, indicating that PfMSH2-1 is involved in recognizing base mispairs and responding to DNA alkylation damage.

## Discussion

HTS revealed many spontaneous mutations that accumulated in MA lines. We were able to construct reliable SNV and indel datasets with low false-negative rates by combining multiple variant callers and manual inspection with IGV [42] (Tables S2 and S7). Indel mutations were more frequent than base substitutions in both Mut and WT. Previous studies have also reported that indels rather than base substitutions are the primary mutations in *P. falciparum* [51, 68]. The mutation rates of WT shown in Table 1 were consistent with those reported previously for *P. falciparum* [8, 53, 68] (Table 1), suggesting that the approach used in this study was reliable.

MSH2 is a protein that plays a critical role in MMR, and its functional deficiency causes increased base substitution and indel mutation rates [21, 69]. In *Plasmodium* species, although the *msh2-2-*disrupted strain has been generated in *P. berghei* NK65 [20], *msh2-1* mutants have never been generated. The P513T mutation in PfMSH2-1 corresponded to the P622T mutation in human MSH2 and the P640T mutation in budding yeast MSH2 (Fig. S1A). The P640T mutation in budding yeast MSH2 increases the mutation rate 198-fold [21]. The P622T mutation in human MSH2 results in folding defects and proteasome-dependent degradation [70]. We considered the possibility that the PfMSH2-1^P513T^ mutant may display changes in the expression of *pfmsh2-1* and its paralogous gene *pfmsh2-2*, but we could not detect any difference in the expression between Mut and WT using real-time RT-PCR (Fig. S1D). The P513T mutation in PfMSH2-1 produced a malarial parasite with an increased indel mutation rate of approximately tenfold and fivefold for STRs and minisatellites, respectively (Table 1), whereas the base substitution rate of Mut was equivalent to that of WT. Previous studies have reported that the consequences of MMR-deficiency on the occurrence of indels and base substitutions vary greatly between taxa, and in particular, MMR-deficiency in eukaryotes results in a far greater increase in the occurrence of small indels than in base substitutions [21, 71–73]. Our results are consistent with these reports; however, it is notable that no statistically significant difference was observed between Mut and WT, although the yeast strain with the corresponding P640T mutation in MSH2 showed a tenfold increase in the base substitution rate [21]. MSH2-1 seems to be able to recognize mispaired nucleotides because the MNNG tolerance test showed that Mut was more resistant to the alkylator than WT (Fig. 4b). These observations suggest that the occurrence of DNA replication errors is limited in the *P. falciparum* genome in the first place, and/or that there is another system to remove mispaired nucleotides, which can compensate PfMSH2-1 defects. Although further studies using knockout/knockdown strains of PfMSH2-1 and/or PfMSH2-2 are necessary to clarify the involvement of PfMSH2-1 in MMR, our results show that PfMSH2-1 plays an important role in suppressing indels and is involved in the recognition of base mispairs resulting from DNA alkylation damage, which eventually induces programmed cell death [74–76].

We used a large indel dataset to investigate the characteristics that affect STR mutability, including length, genomic category and repeat motif type. The indel mutation rate differed depending on the genomic category, and this trend was clearer for Mut than for WT. A higher indel mutation rate was observed in introns than in exons in both Mut and WT (Fig. 1a), and similar results have been reported in wild-type *P. falciparum* 3D7 [53, 68]. We also observed that the mutation rate increased as the size of STRs increased (Fig. 1b). Higher indel mutation rates for STRs with larger allele sizes have been observed in *P. falciparum* [53] and other organisms [77–79]. Notably, the type of repeat motif influences the mutability of STRs. A previous study classified STRs by repeat motif size and compared the indel mutation rates [53]. However, we found that the distribution of STRs in the *P. falciparum* genome was extremely skewed with respect to the motif type (Fig. S4A and Table S8); therefore, discussing mutability by categorizing STRs by motif type would be more appropriate. In Mut, the indel mutation rate in the ‘A’ repeats, which are most abundant in the *P. falciparum* genome, was lower than that in other repeats, such as ‘AT’ and ‘AATAT’ (Fig. 1c). However, it should be noted that the indel mutation rate in the ‘A’ repeats may be underestimated. Unlike the ‘AT’ repeats, the ‘A’ repeats are abundant in exons (Fig. S4), and an indel that occurs there generates a frameshift mutation, which can cause deleterious and sometimes lethal effects. Moreover, the number of MA lines we produced and the number of days for serial *in vitro* culture were limited. Thus, the number of mutations detected in WT was insufficient to conclude a statistically significant difference in the indel mutation rate between repeat motifs using the Tukey–Kramer test; however, ANOVA results showed that the indel mutation rate was significantly different among repeat motifs (*P*<0.05). STR characteristics were found to affect mutability; however, the STR characteristics that have the strongest influence on mutability remain to be analysed. Focusing on the odds ratio (OR) of the random intercept logistic regression model (Table 2), OR was higher for repeat motifs, such as ‘AT’ repeats (OR=14.87, *P*<0.001) and ‘AATAT’ repeats (OR=10.30, *P*<0.001) than for introns (OR=2.16, *P*=0.042), indicating that ‘AT’ and ‘AATAT’ repeat motifs enhanced mutability more than genomic categories. Introns had the highest indel mutation rate (Fig. 1a), and while this is in part because these regions have fewer functional constraints and can thus tolerate indels, it is mainly because they have a high proportion of repeat motif types with a high mutability (Fig. S4). In contrast, the low indel mutation rate in exons is partly due to functional constraints of mutations, but this influence may be small. This appears to be because most repeat motifs in exons have base lengths that are divisible by three (Fig. S4A). The change in the size of STRs in exons generally occurs on a codon-by-codon basis; therefore, indels are unlikely to have a disruptive effect on protein function. Rather, the lower indel mutation rate in exons is due to the low abundance of repeat motifs like ‘AT,’ which enhance mutability. The indel mutation in ‘A’ repeats, which are the second most common in exons (Fig. S4A, B), should have a disruptive effect on protein function caused by a frameshift. Interestingly, mutability in ‘A’ repeats is low in *P. falciparum* (Fig. 1c and Table 2) despite homopolymeric runs known to be hotspots for small indels [73, 80]. The mechanism that suppresses indels in homopolymeric runs is still unknown, but it contributes to the avoidance of frameshift mutations in protein-coding sequences containing ‘A’ repeats.

In Mut, significantly more insertions were detected than deletions (Fig. S5A). Significantly more insertions than deletions have also been observed in the analysis of mitotic ‘clone trees’ generated using the *P. falciparum* field strains [68]. As most of the indels occurred in ‘AT’ repeats (Fig. S5B), indels in ‘AT’ repeats appeared to have the greatest influence on the proportion of insertion and deletion mutations. Analysis of the indel mutations found in ‘AT’ repeats revealed that insertions were more likely to occur when the number of repeats was <20 and that deletions were more likely to occur when the number of repeats was ≥20 (Fig. 2). We may have underestimated the insertion mutation rate in large STRs, because it is difficult to detect small insertions in large STRs using short reads. Despite this, size-dependent mutational bias, in which long alleles are biased toward contraction, while short alleles are biased toward expansion, has been reported [81, 82]. This bias is likely to be ubiquitous across organisms, including *P. falciparum*. The mutability of ‘AT’ repeats is high, but the size-dependent mutational bias may contribute to maintaining the total length of ‘AT’ repeats in the *P. falciparum* genome.

In this study, we observed many HR and telomere healing events in the sub-telomeric regions of the MA clones, and a higher degree of polymorphism was observed in Mut (Fig. 3). PfMSH2-1 is the second example of factors that contribute to the suppression of recombination and maintenance of genome stability in *Plasmodium*, following PfWRN [83]. *P. falciparum* evolutionarily lacks enzymes required for the canonical non-homologous end-joining pathway [2, 84]; therefore, DSBs in sub-telomeric regions are repaired by HR or telomere healing [85]. As the elimination of *cas9* from Mut P0 was confirmed (Fig. S1C), the enhanced instability of sub-telomeric regions in Mut is likely due to the occurrence of more DSBs in Mut than in WT instead of the off-target effect of the residual Cas9. Due to the destabilization of the sub-telomeric regions, we observed deletions of *hrp2* and *hrp3* in Mut. Deletion of these genes affects the performance of RDTs and is a matter of serious concern in malaria control and elimination [86, 87]. Using Mut, we could easily reproduce phenotypes observed in the field in the laboratory. Furthermore, we also observed the generation of chimeric *var* and *stevor,* which seemed to retain their gene structures (Fig. S9D, E). DSBs in the sub-telomeric regions repaired by HR sometimes generate chimeric genes, resulting in the diversification of multicopy variant genes [5, 85]. PfMSH2-1 defects induce more HR events in sub-telomeric regions, which may contribute to antigen diversification and evasion from host immunity. More attention should be paid to MMR defects as a potential risk for destabilization of sub-telomeric regions, which finally creates undesirable outcomes, such as false-negative RDT results and chronic infections.

This study showed that PfMSH2-1 is involved in the maintenance of genome stability by conserving repeat sequences that are abundant in the *P. falciparum* genome and suppressing SVs in sub-telomeric regions. The base substitution rate of Mut was equivalent to that of WT, implying that Mut cannot be used to obtain new mutants by the rapid accumulation of spontaneous base substitutions, such as PbMut [10, 11]. However, Mut may provide extensive antigen diversification in the laboratory, which the successive accumulation of base substitutions cannot achieve. The immune pressure in the host body coupled with the destabilization of sub-telomeric regions may be expected to promote the diversification of antigen genes by HR rather than telomere healing. In addition to the contribution of PfMSH2-1 to genome stability, this study revealed an interesting link between genome instability associated with MMR defects and the evolution of host immune evasion mechanisms.

## Supplementary Data

Supplementary material 1

Supplementary material 2
