# Supplementary material for: Analysis of genome instability and implications for the consequent phenotype in Plasmodium falciparum containing mutated MSH2-1 (P513T)
Source: Microb Genom. 2023 Apr 21;9(4):mgen001003. doi: 10.1099/mgen.0.001003 (PMC10210953; doi:10.1099/mgen.0.001003)
Supplement: Supplementary material 1 [file mgen-9-1003-s001.pdf]

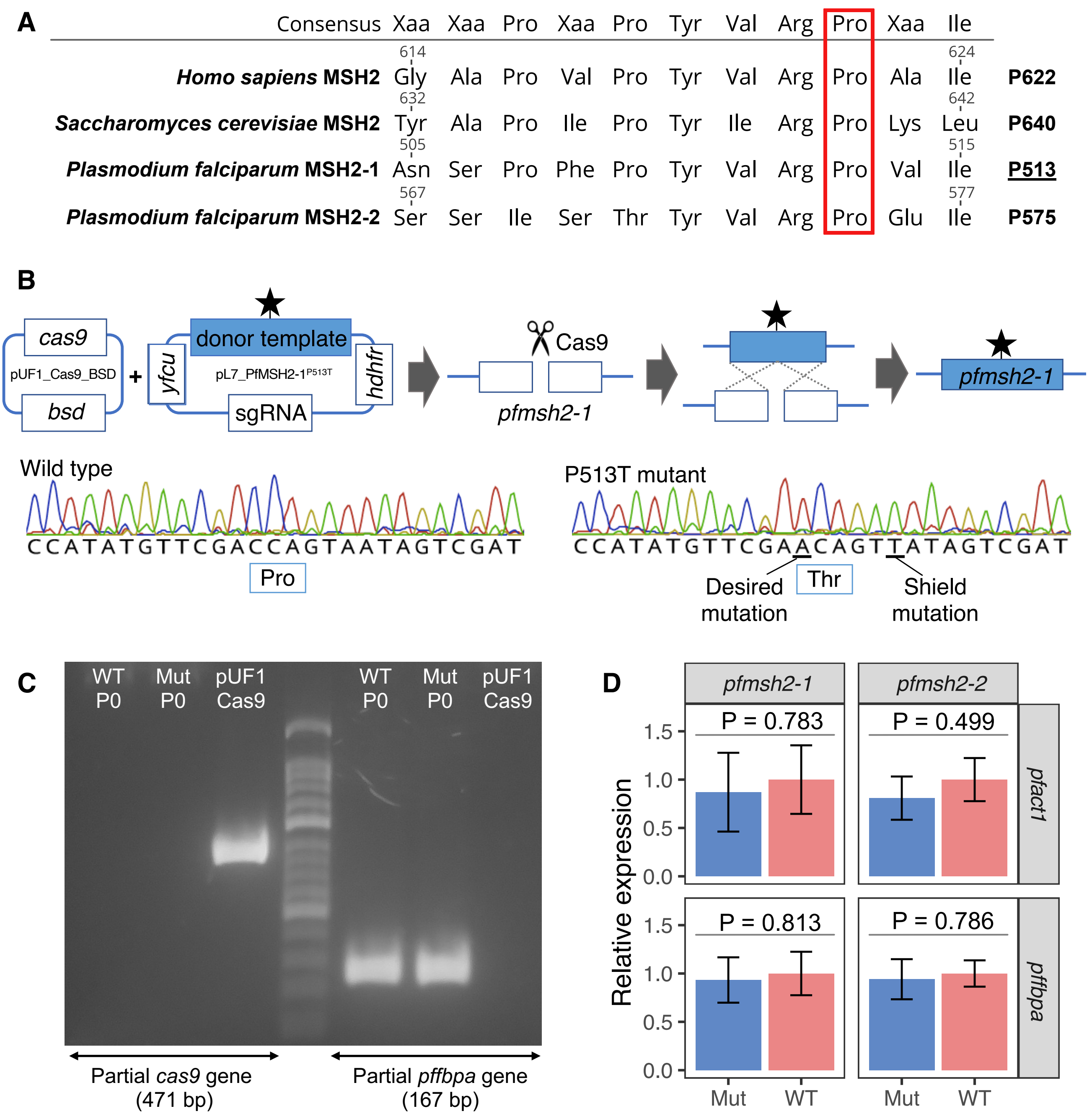

Fig S1. Generation of PfMSH2-1 P513T mutant using the CRISPR/Cas9 technology. (A) Alignment of the amino acid sequences of MSH2 homologs. P622 in human MSH2 and its corresponding amino acid sites in other organisms are framed by a red rectangle. (B) The strategy of CRISPR/Cas9 genome editing. Modified positions of the desired mutation to generate P513T and shield mutation are shown. (C) Confirmatory PCR test with DNA samples (WT P0, Mut P0, and pUF1_Cas9_BSR plasmid) for residual *cas9* gene. No residual *cas9* gene fragment was observed in Mut P0. Amplification of the *pffbpa* gene fragment was performed to confirm PCR functionality. (D) Quantitative real-time PCR assay for estimating gene expression of *pfmsh2-1* and *pfmsh2-2*. We used *pfact1* and *pffbpa* as internal controls. Error bars represent standard error. *P*-values of Welch's *t*-test are shown.

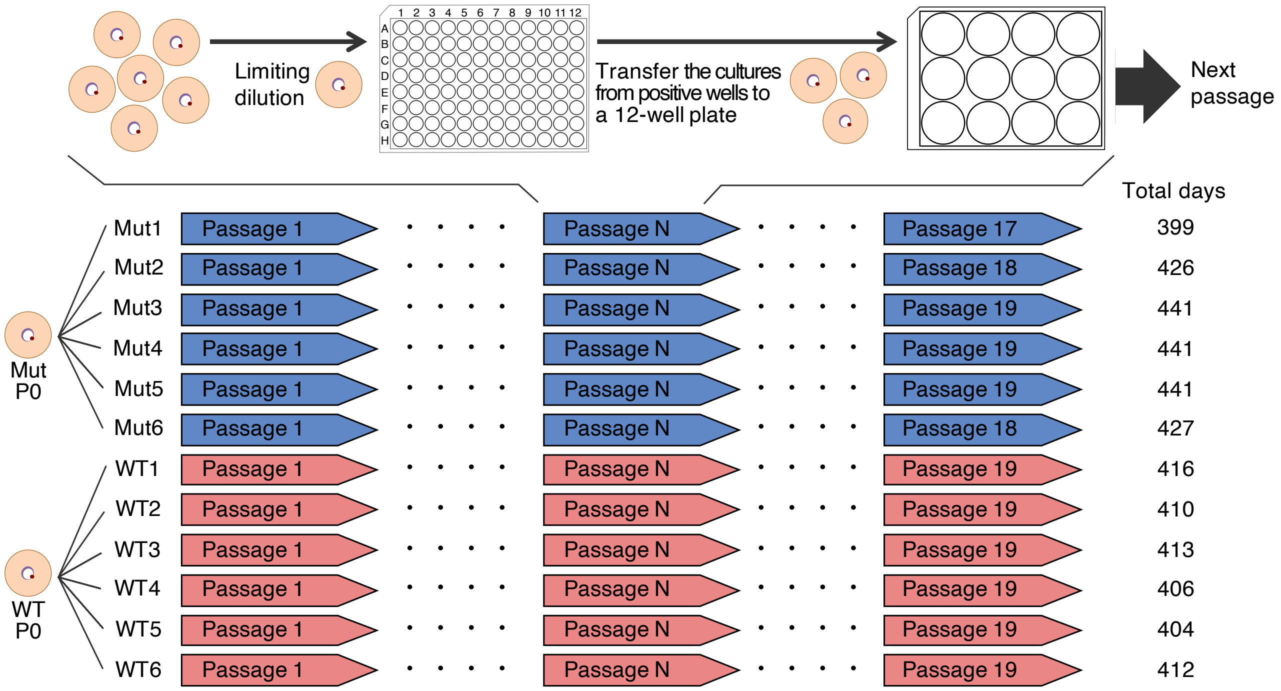

Fig S2. Generation of parallel MA lines with Mut and WT. At the first step of a round of passage, parasites were subjected to limiting dilution and multiplied in a 96-well plate. About two weeks later, a pLDH activity assay was performed to identify positive wells. Cultures were randomly selected from the positive wells, transferred to a 12-well plate, and cultured for an additional approximately five days. One of the cultures for each passage line was subjected to limiting dilution and used for the next round of passage.

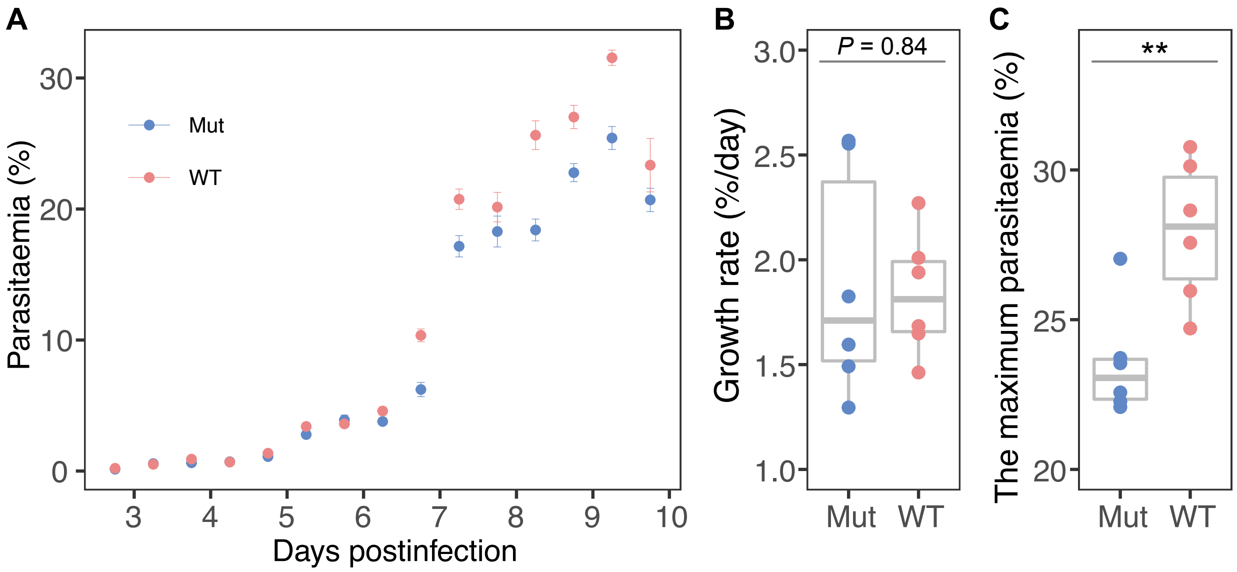

Fig S3. Growth monitoring of Mut and WT clones. (A) Parasitaemia was determined based on the mean of six replicates (± standard error of the means). Error bars represent standard error. The growth data were fitted to the logistic equation to estimate the growth rate (B) and the maximum possible population size, which was considered as the maximum parasitaemia (C). *P*-values of Welch's t-test are shown. $^{**}P < 0.01$.

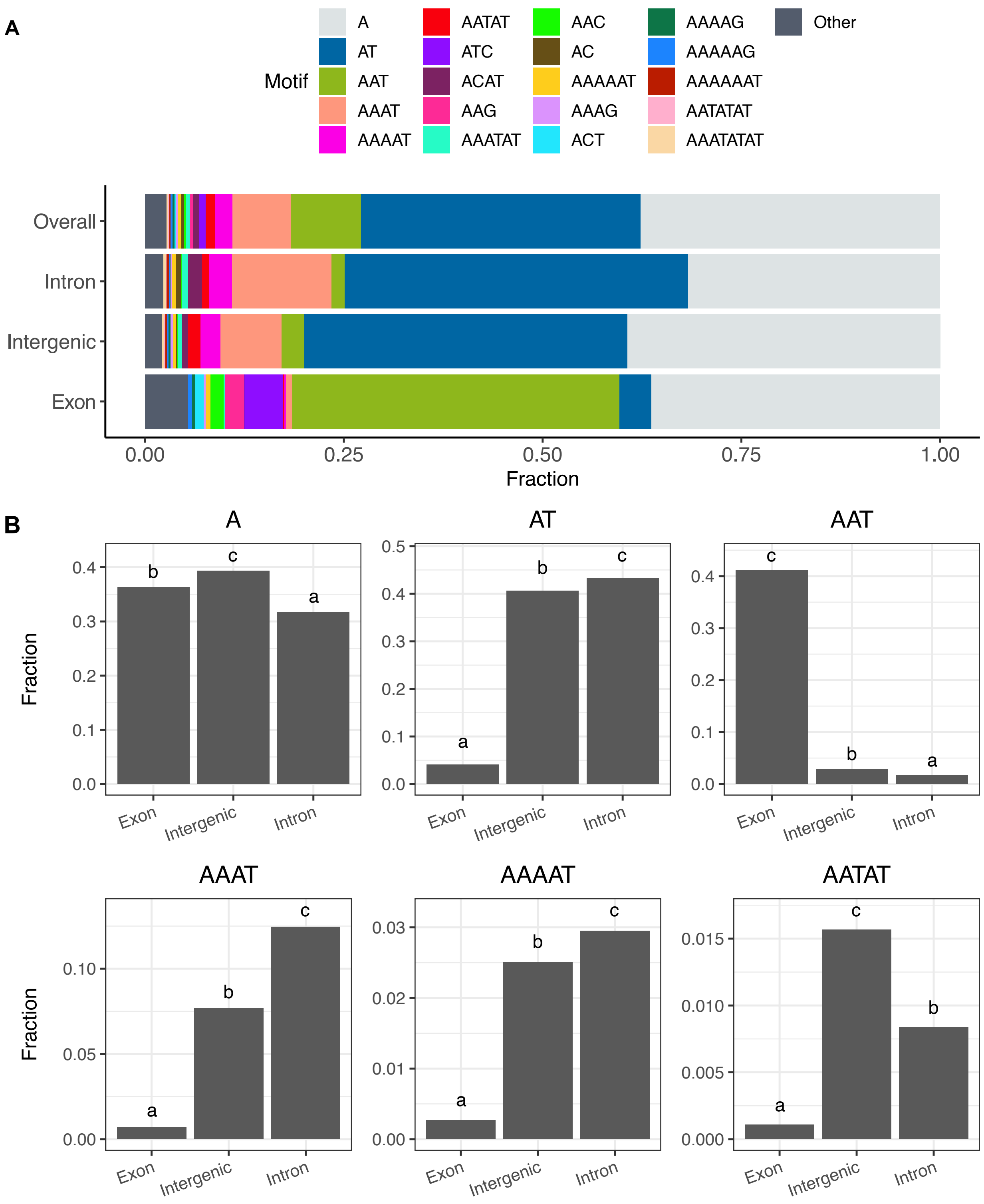

Fig S4. Distribution of STRs in the core genome of *Plasmodium falciparum* 3D7. (A) The fractions of the most abundant 20 STRs and others are shown for exons, introns, intergenic regions, and overall. (B) For the most abundant five repeat motifs, the fractions were compared among exons, introns, and intergenic regions. There was a statistically significant difference among genomic categories with different alphabets (Holm-adjusted $*P < 0.05$; Fisher's exact test).

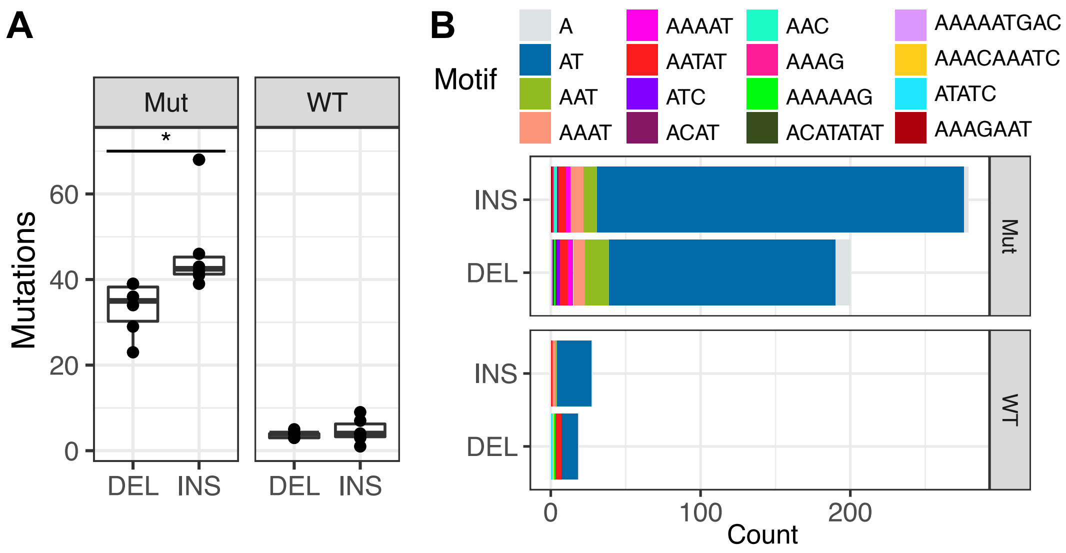

Fig S5. Indel mutations in short tandem repeats. (A) The boxplot shows the number of deletion (DEL) and insertion (INS) mutations for each MA clone. DEL mutations were significantly greater in number than INS mutations in Mut (*$P < 0.05$; Welch's *t*-test). (B) The stacked bar charts show the count of DEL and INS mutations for each repeat motif.

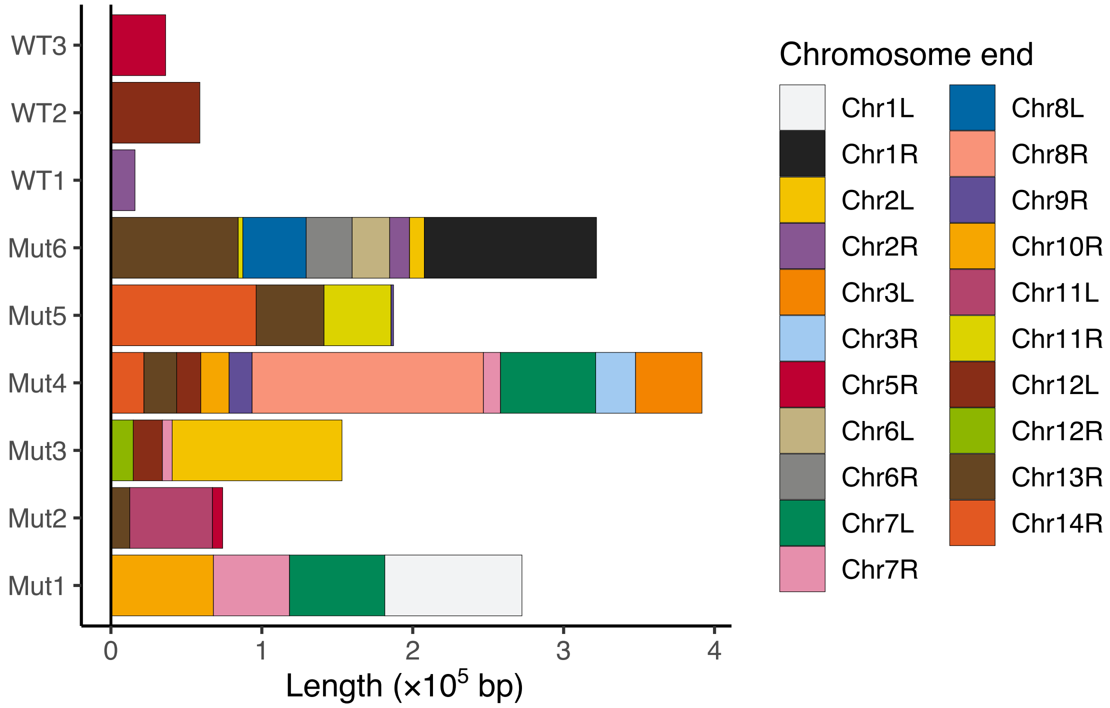

Fig S6. The length of deleted original chromosome ends during mutation accumulation test. The letters "L" and "R" denote the left and right ends of the chromosome, respectively.

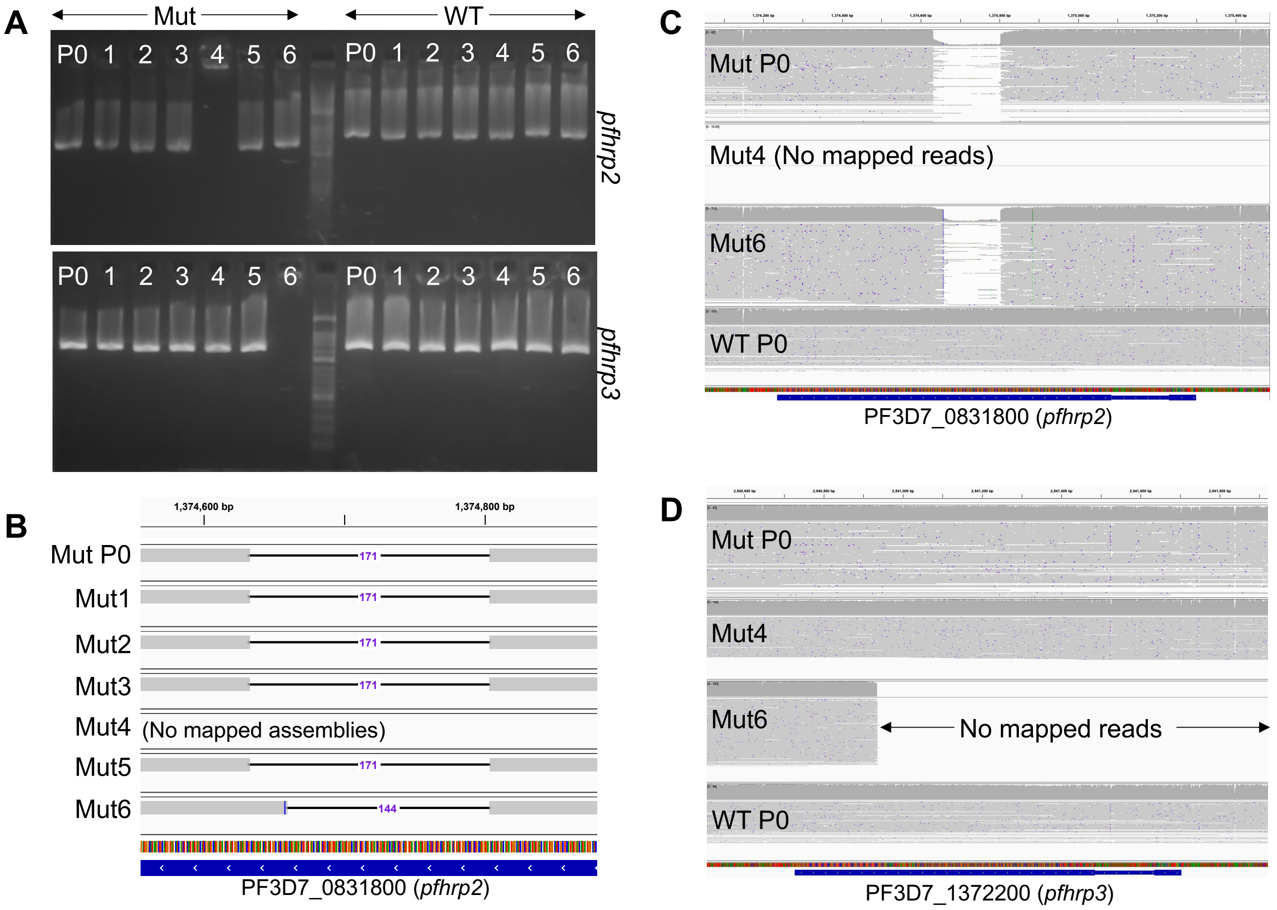

E **Mut P0** CATGGTGGGCATCGTCGTGGTGTGCTCCATCATGGTGGGCATCGTCATGGTGAGAATCAT

Mut6 CATGGTGGGCATCGTCGTGGTGTGCTCCATGGTTCAGGGTTCAGGGTTTAGGGTTCAGGG

Fig S7. Variations in *pfhrp2* and *pfhrp3* genes during the mutation accumulation test. (A) Agarose gel electrophoresis of PCR products amplified from genomic DNA of progenitor and MA clones. PCR reactions targeting *pfhrp2* and *pfhrp3* were performed with primer sets Pfhrp2-F1/Pfhrp2-R1 and Pfhrp3-F1/Pfhrp3-R1, respectively [1]. In *pfhrp2*, no amplification was obtained from Mut4, and the products from other Mut clones were smaller in size than those from WT because Mut P0 already contained a large deletion in the target region, as shown in (B). In *pfhrp3*, no amplification was obtained from Mut6. (B) Mapping Canu assemblies of Mut on the *pfhrp2* gene region. A 171 bp deletion mutation is shared with Mut P0, Mut1, Mut2, Mut3, and Mut5. In Mut4, no assemblies were mapped on the *pfhrp2* region. Mut6 had a 144 bp deletion mutation in *pfhrp2*, indicating that a new 27 bp insertion mutation occurred during long-term passage. (C) Mapping the Oxford Nanopore Technologies (ONT) reads on the *pfhrp2* region. Alignments of Mut P0, Mut4, Mut6, and WT P0 are shown. The result was similar to the alignment result of the assemblies (B). (D) Mapping the ONT reads on the *pfhrp3* region. In Mut6, no reads were mapped to the 5' side of *pfhrp3*. (E) The sequence alignment of the Canu assemblies surrounding the breakpoint of the *pfhrp3* region. Deleted sequence in Mut6 was replaced with telomeric sequence as shown in red letters.

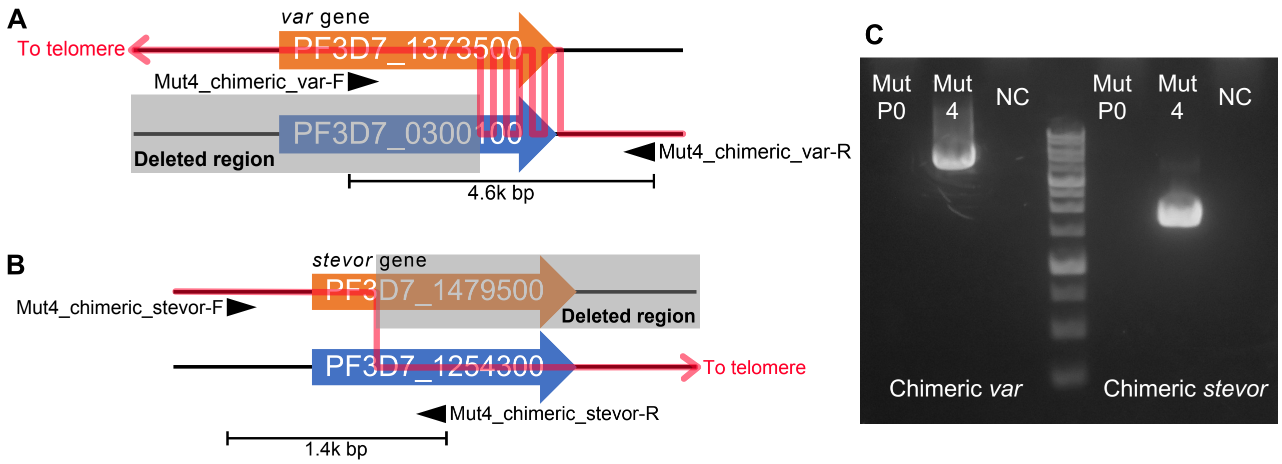

D

······ATGAAGAATTGGATATTAATGACATATATGTACCGCATGCTCCTAAATATAAAACATTGATTGAAGT
GGTACTAGAACCTAGTGGTAACAACACAACAGCTAGTGGTAAGAACACACCAACTAGTGACACACCT
AGTGATACACAAAATGATATACAAAATGATGGTATACCTAGCGATACACCAAATACACCTAGTGATATAC
CAAAAACACCTAGTGATACACCACCACCCATTACTGATGATGAATGGAATACATTGAAAGATGATTTTA
TATCACAATATATACAAAGTGAACAACCAAAGGATGTACCAAATGATTATAGTAGTGGTGATATTCCATT
CAATACACAACCAAACACTTTATATATTGATAATAATCAAGAAAAACCTTTTATTATGTCTATTCATGATA
GAAATTTATATACTGGAGAAGAATACAATTACGATATGAGTAATAATAGTGGTATATATCCAAGTAGTAGT
AACCGTGATTCATTAAGTGGTACAAAAGTTCCATATAGTGGTATAGATTTAATTAATGATTCATTAAATAG
TGGTAATCAACCTATTGATATATATGATGAAGTATTGAAACGAAAAGAAAATGAATTATTTGGGACAAAT
CATGTGAAACAAACAAGTATACATAGTGTTGCCAAACCTGCACGTGACGACCCCCTACACAACCAACT
GGAACTATTCCATAAATGGCTAGATAGACATAGAGATATGTGCGAGAAGTGGAATAATAAAGAGGAACT
ATTAGATAAATTAAAAGAAGAGTGGGAAAATGAGACACATAGTGGTAACACTCACCCTAGTGATAGTA
ACAAAACGTTAAATACTGATGTTTCTATACAGATAGATATGGATGATGGAAAACCAAAGAAGGAATTTA
CTAATATGGATACTATCTTGGAAGATCTGGATAAATATAATGAACCTTATTATGATGTGCAAGATGATATT
TATTATGATGTAAATGATCATGATGCATCAACTGTGGATACTAATGCTATGGATGTTCCTAGTAAAGTACA
AATTGAAATGGATGTAAATACCAAATTGGTGAAAGAGAAATATCCTATAGCAGATGTATGGGATATTTAA

E

ATGAAGATGTATTATCTAAAAATGTTATTGTTTACCTTTTTAATAAATACATTAATATTACCACATTATGTAT
GTAAAAAAGATGCATTATTATTACAATAACGTAAATATATAATTAAACAATTATTACATTTTCAGTACATAT
TGATTAATCTTCTTTTTCTTCTTTTAGGAGAATTATCTAAATAATGATTATAATGTAAGTTTCATTCAAAAC
AAGACCAAAAGAGCAACGATAAAATCAAGACGTTTAGCACAAACCCAAATCCATAATCCGCATTATCA
TAATGATCCAGAACTCAAAGAAATAATTGATAAAATGAACGAAGAAGCAATTAAAAAATACCAAAAATC
TCATGATCCATATGAACAATTAAAAGAAGTAGTAGAAAAAAATGGAACAAAATATACAGGTGGTAATGA
TGCAAAACCTATGTCAGCGATAGAAAAAGAATTATTGAAAACATATGAAGAAAGGTTTGGTAACG······

Fig S8. Mitotic recombination events and the generation of chimeric genes in Mut4. (A) Schematic view of the binding between PF3D7_0300100 (blue) and PF3D7_1373500 (orange), leading to the generation of a new chimeric *var* gene. The triangles Mut4_chimeric_var-F and Mut4_chimeric_var-R represent the positions of primers to test the binding. (B) Schematic view of the binding between PF3D7_1479500 (orange) and PF3D7_1254300 (blue), leading to the generation of a new chimeric *stevor* gene. The triangles Mut4_chimeric_stevor-F and Mut4_chimeric_stevor-R represent the positions of primers to test the binding. (C) Confirmatory PCR test with genomic DNA of Mut P0, Mut4, and distilled water as the negative control (NC). PCR reactions were performed using primers displayed in (A) and (B). Positive PCR results were obtained from only Mut4. (D) The sequence of the chimeric *var* gene was determined using Sanger sequencing. The chimeric *var* gene consists of sequences from PF3D7_1373500 (orange) and PF3D7_0300100 (blue). The TAA stop codon is underlined. (E) The sequence of the chimeric *stevor* gene was determined using Sanger sequencing. The chimeric *stevor* gene consists of sequences from PF3D7_1479500 (orange) and PF3D7_1254300 (blue). The ATG start codon is underlined. Bases that are identical between the two genes are highlighted in grey.

REFERENCE

1. **Baker J, McCarthy J, Gatton M, Kyle DE, Belizario V et al.** Genetic diversity of *Plasmodium falciparum* histidine-rich protein 2 (PfHRP2) and its effect on the performance of PfHRP2-based rapid diagnostic tests. *Journal of Infectious Diseases* 2005;192(5):870-877.
